# Anti-HIV-1 Activity of Elafin Depends on Its Nuclear Localization and Altered Innate Immune Activation in Female Genital Epithelial Cells

**DOI:** 10.1371/journal.pone.0052738

**Published:** 2012-12-27

**Authors:** Anna G. Drannik, Kakon Nag, Xiao-Dan Yao, Bethany M. Henrick, T. Blake Ball, Francis A. Plummer, Charles Wachihi, Joshua Kimani, Kenneth L. Rosenthal

**Affiliations:** 1 Department of Pathology and Molecular Medicine, McMaster Immunology Research Centre, Michael G. DeGroote Institute for Infectious Disease Research, McMaster University, Hamilton, Ontario, Canada; 2 Department of Medical Microbiology, University of Manitoba and Public Health Agency of Canada, Winnipeg, Manitoba, Canada; 3 Department of Medical Microbiology, University of Nairobi, Nairobi, Kenya; University of Central Florida College of Medicine, United States of America

## Abstract

Elafin (E) and its precursor trappin-2 (Tr) are alarm antiproteases with antimicrobial and immunomodulatory activities. Tr and E (Tr/E) have been associated with HIV-1 resistance. We recently showed that Tr/E reduced IL-8 secretion and NF-κB activation in response to a mimic of viral dsRNA and contributed to anti-HIV activity of cervicovaginal lavage fluid (CVL) of HIV-resistant (HIV-R) commercial sex workers (CSWs). Additionally, Tr, and more so E, were found to inhibit attachment/entry and transcytosis of HIV-1 in human endometrial HEC-1A cells, acting through virus or cells. Given their immunomodulatory activity, we hypothesized that Tr/E could exert anti-HIV-1 activity at multiple levels. Here, using tagged and untagged Tr/E proteins, we comparatively evaluated their protease inhibitory, anti-HIV-1, and immunomodulatory activities, and cellular distribution. E appeared to function as an autocrine/paracrine factor in HEC-1A cells, and anti-HIV-1 activity of E depended on its unmodified N-terminus and altered cellular innate activation, but not its antiprotease activity. Specifically, exogenously added N-terminus-unmodified E was able to enter the nucleus and to reduce viral attachment/entry and transcytosis, preferentially affecting R5-HIV-1_ADA_, but not X4-HIV-1_IIIB_. Further, anti-HIV-1 activity of E was associated with significantly decreased HIV-1-triggered IL-8 release, attenuated NF-κB/p65 nuclear translocation, and significantly modulated mRNA expression of innate sensors TLR3 and RIG-I in HEC-1A cells. Most importantly, we found that elevated Tr/E in CVLs of HIV-R CSWs were associated with lower mRNA levels of TLRs 2, 3, 4 and RIG-I in the genital ECs from this cohort, suggesting a link between Tr/E, HIV-1 resistance and modulated innate viral recognition in the female genital mucosa. Collectively, our data indicate that unmodified N-terminus is critical for intranuclear localization and anti-HIV-1 activity of E. We also propose that E-mediated altered cellular innate activation most likely contributes to the HIV-R phenotype of these subjects.

## Introduction

Genital epithelial cells (ECs) are primary sentinels in the female genital tract (FGT) [1], [2]. ECs express a number of pattern-recognition receptors (PRRs) involved in viral recognition, including Toll-like receptors (TLRs) 2, 3, 4, 7, 8 and 9 and RNA helicases, retinoic acid inducible gene (RIG)-I and melanoma-differentiation-associated gene 5 (MDA5) [3], [4], [5], [6]. Sensing of viruses, including HIV-1, through PRRs initiates a series of signal transduction events that activate key transcription factors, including nuclear factor-kappa B (NF-κB) [7], [8], that induce innate [9], [10], [11], [12] and adaptive [13], [14] immune responses. Such responses, however, have also been implicated in HIV/AIDS pathogenesis and disease progression through chronic immune activation, progressive cell loss, and ultimately inability to clear the pathogen [15], [16], [17], [18]. Thus, factors or measures actively controlling immune activation while augmenting antiviral protection might be beneficial in altering the course of HIV-1. While microbicides and vaccines still hold promise to control HIV-1 infection [19], [20], their success clearly depends on understanding of factors and early events in host/virus interaction that allow or divert the establishment of infection at the portal of viral entry. Hence, studies conducted in highly HIV-exposed seronegative (HESN) individuals that remain free of infection and disease can shed light on the correlates and events determining resistance against HIV-1 in the female genital mucosa [21]. Recently, serine protease inhibitors trappin-2 (Tr) and elafin (E) have been associated with HIV-1 resistance in HESN individuals [22].

Tr and E (Tr/E) are alarm antiproteases and mucosal regulators of immunity with well documented antimicrobial, immunomodulatory, and tissue repair properties [23], [24]. Tr/E are members of the whey acidic protein (WAP) family that contain a characteristic and evolutionary conserved four-disulfide core (FDC), or WAP domain, involved in protease inhibition [25], [26]. Tr (9.9 kDa) is a secreted precursor molecule that has two domains. In its N-terminal domain, Tr contains a transglutaminase substrate-binding domain (TSBD), allowing coupling of Tr to extracellular matrix proteins [27]. Proteolytic cleavage of the N-terminus of Tr produces elafin (E) (5.9 kDa), containing a WAP inhibitory domain [28] and one TSBD [29].

Similar to their structural homolog, secretory leukocyte protease inhibitor (SLPI) [26], Tr/E possess antimicrobial activity against bacteria, fungi, and viruses, including HIV-1 [11], [23], [30], [31]. Several mechanisms of Tr/E antimicrobial activity have been proposed, including their direct interaction with microbial cell membrane due to the cationic nature of these molecules [31], bacterial opsonization [32], and binding to bacterial DNA [33]. Tr/E were also shown to possess immunomodulatory properties [24]. E was shown to inhibit LPS-triggered release of MCP-1 in monocytes [34], whereas Tr/E reduced IL-8 and TNFα secretion in response to human elastase, LPS, and oxidized LDL in endothelial cells and macrophages TNF [35], [36]. This immunomodulation has been attributed to binding to LPS [35] or inhibition of NF-κB and AP-1 pathways activation [34], . These findings demonstrate the pleiotropic nature of Tr/E and their contribution to host defense at differential levels: targeting both pathogens and host's immune-inflammatory responses.

Tr/E have also been linked to inflammatory disorders of lung [37], [38], gut [39] and skin [25]. Tr/E are constitutively expressed in mucosal secretions [9], [11] and can be induced in response to IL-1β, TNFα, and polyI:C [3], [11], [40]. Tr/E are produced by multiple cell types, including genital ECs [3], [11], [40] and have been identified in cervicovaginal lavage (CVL) fluid [22], [30]. Together with SLPI and a plethora of other effector molecules, namely defensins, serpins, cystatins, lysozyme, and lactoferrin, Tr/E are believed to play a significant role in protecting the FGT against STIs, including HIV-1 [9], [11], [12], [30], [41], [42].

Recently, we and others demonstrated that both Tr and E possessed antiviral activity against VSV-GFP [3], HSV-2 (Drannik *et al.*, in revision in J. Virol.), and HIV-1 [11], [30]. Anti-HIV-1 activity of Tr/E appeared to be cell type and virus strain dependent [30]. We further found that anti-HIV activity of E was approximately 130 times more potent than Tr, and that both Tr/E acted *via* virus and cells in reducing attachment and transcytosis of R5-HIV-1_ADA_ through endometrial cells lacking canonical HIV-1 receptors (HEC-1A). We confirmed the presence of higher levels of Tr/E in CVL of HIV-1 resistant (HIV-R) commercial sex workers (CSW) and found that Tr/E accounted for approximately 60% of the natural anti-HIV-1 activity of CVL in *in vitro* assays [30]. Additionally, we recently showed that Tr/E reduced IL-8 and TNFα secretion and activation of NF-κB in HEC-1A cells in response to polyI:C [3], suggesting that Tr/E can act both through virus and cells in their antiviral activity. Taken together, these findings demonstrate the significance of E as an anti-HIV-1 molecule and warrant determining the parameters and mechanisms of E's anti-HIV-1 action, including its ability to modulate HIV-induced innate immune responses. In this study, we show that E-mediated decrease in HIV-1 attachment/entry and transcytosis is associated with its N-terminus, intranuclear localization and accompanied by reduced IL-8 secretion, attenuated NF-κB activation, and lower expression of innate viral sensors.

## Materials and Methods

### Reagents and cell lines

Polyinosinic/polycytidylic acid (polyI:C) (Sigma-Aldrich, Oakville, ON, Canada) was reconstituted in PBS at indicated concentration. Human endometrial carcinoma (HEC-1A, ATCC # HTB-112™, deposited by Dr. H. Kuramoto) and TZM-bl (JC53-BL) (ATCC # PTA-5659, U.S. Patent Number 6,797,462, contributed by Drs. John Kappes and Xiaoyun Wu) cells were obtained from American Type Culture Collection (ATCC) (Rockville, MD, USA) and maintained in McCoy's 5A Medium Modified (Invitrogen Life Technologies, Burlington, Ontario, Canada) and DMEM, respectively, supplemented with 10% fetal bovine serum, 1% HEPES (Invitrogen Life Technologies), 1% l-glutamine (Invitrogen Life Technologies), and 1% penicillin-streptomycin (Sigma-Aldrich, Oakville, Ontario, Canada) at 37°C in 5% CO_2_.

### Protease inhibition assay

Elastase-inhibitory activity was measured as described previously [30], [43]. Briefly, Tr/E protein (final volume 10 µl/well), or diluent alone, was combined in a 96-well plate with 50 ng in 10 µl/well of purified human neutrophil elastase (HNE) (Sigma-Aldrich) or diluent alone (negative control), and incubated for 30 min at 37°C. Subsequently, 50 µl of HNE substrate, *N*-methoxysuccinyl-Ala-Ala-Pro-Val *p*-nitroanilide (Sigma-Aldrich), diluted to 50 µg/ml in buffer (50 mM Tris, 0.1% Triton, 0.5 M sodium chloride, pH 8) was added. The hydrolysis was measured by monitoring the absorbance at 405 nm for 15 min using a Tecan Safire ELISA reader (MTX Labs Systems).

### Study participants

The study participants were described in more detail elsewhere [30]. Briefly, women within a cohort of Pumwani CSWs from Nairobi, Kenya, were enrolled during scheduled biannual resurveys in two study groups: HIV-R, HIV-S. This is an ongoing, open cohort with participants enrolled between years 1989 and 2009. Within the cohort, women who remained HIV negative for 7 years of follow-up, as assessed by both serology and RT-PCR, and who were clinically healthy and free of concomitant sexually-transmitted infections (STIs) as well as remained active in sex work were considered relatively HIV-resistant HIV-R [44]_. Participants, who were HIV-uninfected CSWs but had been followed up for less than 7 years, were defined as HIV-S. All the participants in the cohort had similar socio-economic and genetic backgrounds. No CSWs enrolled in this study were found to have co-existing STIs. Study protocols were approved by ethics review boards at the Universities of Nairobi, Manitoba, and McMaster. All participants provided signed, informed consent.

### Isolation of mucosal samples

Cervical EC samples were collected using scraper and endocervical cytobrush following standard protocol. The EC cells were purified from the scraped materials using a standard Ficoll gravitational protocol, which were dissolved in 0.8 ml of TRIzol and stored at −70°C. Cervical ECs were available for each of the groups as follows: (HIV-S, N = 10 and HIV-R, N = 10).

### Trappin-2 (Tr) and elafin (E) proteins for in vitro experiments

The following Tr and E protein preparations were used in this study: human commercial recombinant Tr (cTr) (with a C-terminus His-tag) (R&D Systems, Burlington, ON, Canada) [30], which is a mixture of Tr and E [3]; secreted trappin-2 (sTr) (without a tag) that was generated following infection of HEC-1A cells with a replication-deficient adenovirus vector encoding gene for the human Tr, as described elsewhere [3]; human commercial recombinant elafin (E) (without a tag) HC4011 (Hycult Biotech, Uden, Netherlands); in-house human recombinant HAT-E (hE) (with an N-terminus tag) that was generated as described earlier [3]; the C-terminally 6×His-tagged E (Eh) was purified from cTr by immunodepletion of Tr using cyanogen bromide (CNBr) beads, as per supplier's protocol (Sigma-Aldrich), that were chemically conjugated to goat-anti-Tr/E antibodies, raised against the N-terminus of Tr (AF1747, R&D Systems). Antibody-conjugated beads were prepared as described previously [30], and loaded into a column. The cTr was reconstituted in PBS and slowly passed through the column for 10 times at 4°C. Flow through (Eh) was collected and concentrated by sequentially using 30 kDa and 3 kDa molecular cut-off (Millipore) microcentrifugation tubes. Samples were aliquoted and stored at −80°C, and used as necessary. Due to expensive preparation and thus sample limitation, Eh was used only in limited comparison experiments with cTr as well as in confocal microscopy. Concentrations of Tr and E used in this study were based on a dose-dependent anti-HIV-1 activity of the proteins that was reported earlier [30].

### HIV-1 stock preparation and related assays

The lab-adapted HIV-1_IIIB_ strain (X4-tropic) was prepared in PBMCs and HIV-1_ADA_ virus (R5-tropic) was prepared in adherent macrophages purified from human PBMC as was described elsewhere [45]. A dose of 10 ng of p24 was used for transcytosis and immunofluorescence experiments. Median tissue culture infectious dose (TCID_50_) for each final stock was determined using the Reed-Muench method in TZM-bl cells (5.00×10^3^/ml for X4-HIV-1_IIIB_ and 2.77×10^4^/ml for the R5-HIV-1_ADA_ stock); 100 TCID_50_ of each HIV-1 stocks were used in HIV-1 attachment assays that represented 860 pg for X4-HIV-1_IIIB_ and 504 pg for R5-HIV-1_ADA_ stock.

### HIV-1 attachment/entry and transcytosis in HEC-1A cells

HIV-1 attachment/entry and transcytosis assays were performed as described previously in references [30], [46], [47], with slight modifications. For attachment/entry assay, 100 TCID_50_ of R5-HIV-1_ADA_, X4-HIV-1_IIIB_ or HEC-1A cells, grown in a 96-well plate to full confluence, were individually incubated with media alone or tested Tr/E proteins at 1 µg/ml for 1 h at 37°C to see separate anti-HIV-1 effect mediated through virus and cells. Following the incubation, cells were repeatedly washed with PBS. Subsequently, cells not pretreated with the proteins received either medium (−), untreated HIV-1 (V), or HIV-1 preincubated with Tr or E (V+p) for another 1.5 h at 37°C. Cells initially pretreated with the proteins (c+p) received untreated HIV-1 alone. Following 1.5 h of incubation, viral inoculum was removed, cells were repeatedly washed (4×) and lysed (1% Triton X-100 for 45 min at 37°C). Cell lysates were harvested and centrifuged at 11,000×*g* for 5 min. The amount of total cell lysate-associated p24 was determined by ELISA and expressed as pg/ml.

Transcytosis assay was described previously in reference [30]. Briefly, 1×10^5^ HEC-1A cells were seeded per insert and grown as a tight polarized monolayer on a permeable polycarbonate support (0.4-µm pore-diameter membrane tissue culture inserts, BD Falcon, Mississauga, Canada) over minimum of 3 days. Cells that reached transepithelial resistance (TER) 350–470Ω/cm^2^ (EVOM; World Precision Instruments, Sarasota, FL, USA) were considered confluent and were pretreated apically with media or 1 µg/ml of Tr or E proteins for 1 h before the addition of medium or 10 ng p24 of R5-HIV-1_ADA_ or X4-HIV-1_IIIB_ for 8 h at 37°C to the apical chamber. Amount of infectious HIV-1 particles in the basolateral chamber were determined using TZM-bl cells as described elsewhere [46], with slight modifications. Briefly, basolateral supernatants were concentrated in 30-K Amicon microcentrifugation tubes (Millipore) and quantitatively analyzed by testing β-galactosidase activity of TZM-bl cells, resulting in cells turning blue upon HIV-1 infection [46]. TZM-bl indicator cells were plated in 24-well plates at 6×10^4^ cells per well in complete growth medium the day before infection. The cells were infected by adding to each well dilutions of virus in a total volume of 150 µl in the presence of 20 µg/ml of DEAE-dextran (Sigma). The plates were rocked every 30 to 45 min, and 1 ml of complete growth medium per well was added after 2 hours. Two days later, the cells were fixed at room temperature with 2 ml of a solution of 1% formaldehyde-0.2% glutaraldehyde in phosphate-buffered saline (PBS) for 5 min.

The cells were then washed three times with PBS and incubated for 50–60 min at 37°C in 300 µl of a solution of 4 mM potassium ferrocyanide, 4 mM potassium ferricyanide, 2 mM MgCl2, and 0.4 mg of X-Gal per ml. The reaction was stopped by removing the staining solution and washing the cells twice with PBS, and blue cells were counted under a microscope. Data are expressed as percentage of infectious particles recovered in the presence of Tr or E compared to the percentage of virus recovered in the virus control alone, taken as 100%. Concentrations of Tr and E used in these experiments were based on a dose-dependent anti-HIV-1 activity of the proteins that was reported earlier [30].

### MTT viability assay

MTT assay (Biotium Inc., Hayward, CA, USA) was used as per manufacturer's instructions to determine viability of HEC-1A cells and was described elsewhere [5], [30]_.

### ELISA assays

CVLs and cell-free supernatants of HEC-1A cells were stored at −70°C until assayed for human Tr/E, IL-8, and TNF-α with ELISA Duoset kit (R&D Systems) according to the supplier's protocol. Cut off limit for Tr/E and IL-8 was 31.25 pg/ml; TNF-α 15.6 pg/ml. p24 was detected by HIV-p24 ELISA as per supplier's protocol (HIV-1 p24 Antigen Capture Assay, Advanced BioScience Laboratories, Inc., Kensington, MD, USA) with a cut off limit of 3.1 pg/ml. Analytes were quantified based on standard curves obtained using an ELISA reader Tecan Safire ELISA reader (MTX Labs Systems Inc.).

### Immunofluorescence staining

Immunofluorescence staining was performed as described elsewhere [3], [5]_, but with minor modifications. HEC-1A cells grown on an 8-well BD Falcon culture slides (BD Biosciences) were pretreated with various Tr and E protein preparations, for 1 h or with polyI:C (25 µg/ml per well), or 10 ng HIV-1 p24 for 4 h. Following the above treatments, cells were fixed and blocked as described previously. _NF-κB p65 sc-372 (SantaCruz Biotechnologies, Santa Cruz, CA, USA) (1∶500) were used to detect nuclear translocation of NF-κB p65. Negative control rabbit immunoglobulin fraction (DakoCytomation, Glostrup, Denmark) was used as an isotype control. TRAB2O (HM2062, Hycult Biotech, Uden, Netherlands) primary antibodies were used to detect Tr/E. HAT and 6×His tags were detected using anti-HAT (LS-C51508, LifeSpan Biosciences, Inc., Seattle, WA, USA), and anti-His (#2365, Cell Signaling, Denvers, MA, USA) antibodies, respectively. Corresponding Alexa Fluor 488 conjugated IgG (Molecular Probes, Eugene, OR, USA) were used as secondary antibody. Nuclei were visualized by staining with propidium iodide. Images were acquired using an inverted laser-scanning confocal microscope (LSM 510, Zeiss, Oberkochen, Germany).

### RNA extraction and real-time quantitative PCR (RT-qPCR) analysis

Total RNA was isolated from cervical ECs and HEC-1A cells using TRIzol (Invitrogen Life Technologies). DNase-treated (Ambion, Austin, TX, USA), total RNA was reverse transcribed with SuperScript reverse transcriptase III (Invitrogen Life Technologies). RT-qPCR was performed in a total volume of 25 µl using Universal PCR Master Mix (Applied Biosystems, Foster City, CA, USA), and TaqMan protocol. The following primers were used: RIG-I: 5′-AGGGCTTTACAAATCCTGCTCTCTTCA-3′ (probe), 5′-GGTGTTCCAGATGCCAGAC-3′ (forward), 5′-TTCCGCAAATGTGAAGTGTATAA-3′(reverse); MDA5: 5′-TTTGGCTTGCTTCGTGGCCC-3′(probe), 5′-TGATTCCCCTTCCTCAGATAC-3′(forward), 5′-TGCATCAAGATTGGCACATAGT-3′(reverse); TLR2: 5′- TCCTGCTGATCCTGC-3′ (probe), 5′- TGGCATGTGCTGTGCTCTG-3′ (forward), 5′- GGAAACGGTGGCACAGGAC-3′ (reverse); TLR3: 5′-TGTGGATAGCTCTCC-3′(probe), 5′-CCGAAGGGTGGCCCTTA-3′(forward), 5′-AAGTTACGAAGAGGCTGGAATGG-3′ (reverse); TLR4: 5′- TGTGCAACACCTTCAG-3′ (probe), 5′- GGTGGAAGTTGAACGAATGGA-3′ (forward), 5′- AACTCAGCACAGGCATGCC-3′ (reverse); TLR7: 5′-ATTCTCCCCTACAGATC-3′ (probe), 5′-TTCCTTGTGCGCCGTGTAA-3′ (forward), 5′-TCAGCGCATCAAAAGCATTTA-3′ (reverse); TLR8: 5′-ATGAGCTGCGCTACC-3′ (probe), 5′-GCCTCTGTTACTGACTGGGTGAT-3′ (forward), 5′- TTGTCTCGGCTCTCTTCAAGG-3′ (reverse); 18S rRNA: 5′-CGGAATTAACCAGACAAATCGCTCCA -3′ (probe), 5′-GTGCATGGCCGTTCTTAGTT-3′ (forward), 5′-TGCCAGAGTCTCGTTCGTTAT-3′ (reverse). The expression of 18S ribosomal RNA was used as an internal control. PCRs were performed with an ABI PRIZM 7900HT Sequence Detection System using the Sequence Detector Software 2.2 (Applied Biosystems).

### Statistical analysis

Data were expressed as means ± standard deviation (SD). Statistical analysis was performed with either non-paired Student's *t* test or a one-way analysis of variance (ANOVA) using Sigma Stat 2.0.

## Results

### N-terminus of elafin is critical for anti-HIV-1, but not protease inhibitory activity

**Figure 1:**
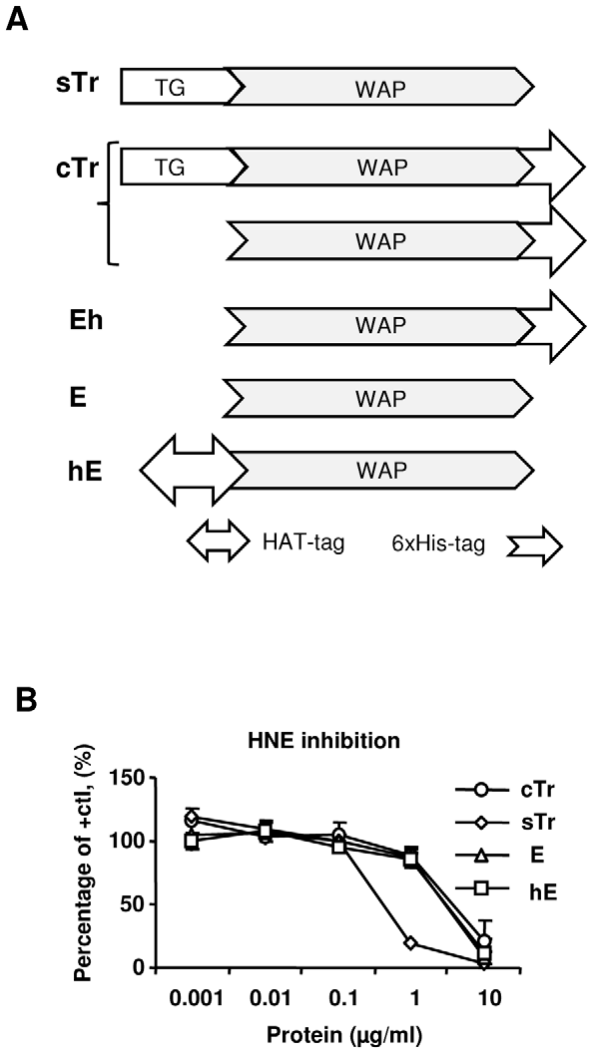
Schematic and protease inhibitory properties of trappin-2 and elafin protein preparations. (*A*) Schematic diagrams of tested human trappin-2 (Tr) and elafin (E) (Tr/E) protein preparations. sTr, human secreted Tr (without a tag); TG, transglutaminase domain; WAP, whey acidic protein domain; cTr, human commercial recombinant 6×His-trappin-2, a mixture of both Tr and E (with a C terminus His-tag); Eh, human recombinant E purified from cTr (with a C terminus His-tag); E, human commercial recombinant elafin (without a tag); hE, in-house human recombinant HAT-E (with an N terminus HAT-tag). (*B*) Protease inhibitory activity of different Tr/E proteins was tested by combining human neutrophil elastase (HNE) with various concentrations of Tr or E and measuring residual activity of HNE by adding a chromogenic elastase specific substrate *N*-methoxysuccinyl-Ala-Ala-Pro-Val *p*-nitroanilide and monitoring the change in absorbance at 405 nm over 15 min. Results are expressed relative to the NHE activity of a positive control (HNE in diluent alone); cTr (○); sTr, (◊); E, (▵); hE, (□).

To determine the parameters of anti-HIV-1 activity of E and to test the hypothesis that anti-HIV-1 effect of E might be associated with immunomodulation, we employed several Tr/E proteins that were comparatively analyzed for anti-HIV-1 activity. Figure 1A provides schematics of various trappin-2 (Tr) and elafin (E) proteins tested in this study, including: human commercial Tr - a mixture of both Tr and E with a C-terminal His-tag (cTr); human secreted Tr without a tag (sTr), used as a control for cTr; human commercial E without a tag (E); human E purified from cTr with a C-terminal tag (Eh); and human in-house E with an N-terminal tag (hE). Protease inhibitory activity is a major function of Tr/E [26] and can reflect the proteins' functional integrity. Here, similar to our previous reports [3], [30], protease inhibitory activity of utilized Tr/E proteins was evaluated to test functional integrity and ensure that different manufacturing sources and preparations of Tr/E proteins did not interfere with their function. Tr/E proteins were incubated with HNE for 30 min at 37°C, and residual activity of HNE was determined by adding a chromogenic elastase specific substrate and monitoring the change in absorbance over time. Results of the protease inhibitory assay indicated that all the tested proteins were equipotently active as protease inhibitors at 10 µg/ml concentration (Fig. 1B). Eh preparation was not evaluated for antiprotease function due to sample limitation. We also previously demonstrated that cTr, sTr, and hE were comparable in their ability to modulate polyI:C-induced IL-8 secretion by HEC-1A cells [3], thus confirming that different sources did not significantly influence the proteins' functions in the context of human elastase and polyI:C.

**Figure 2:**
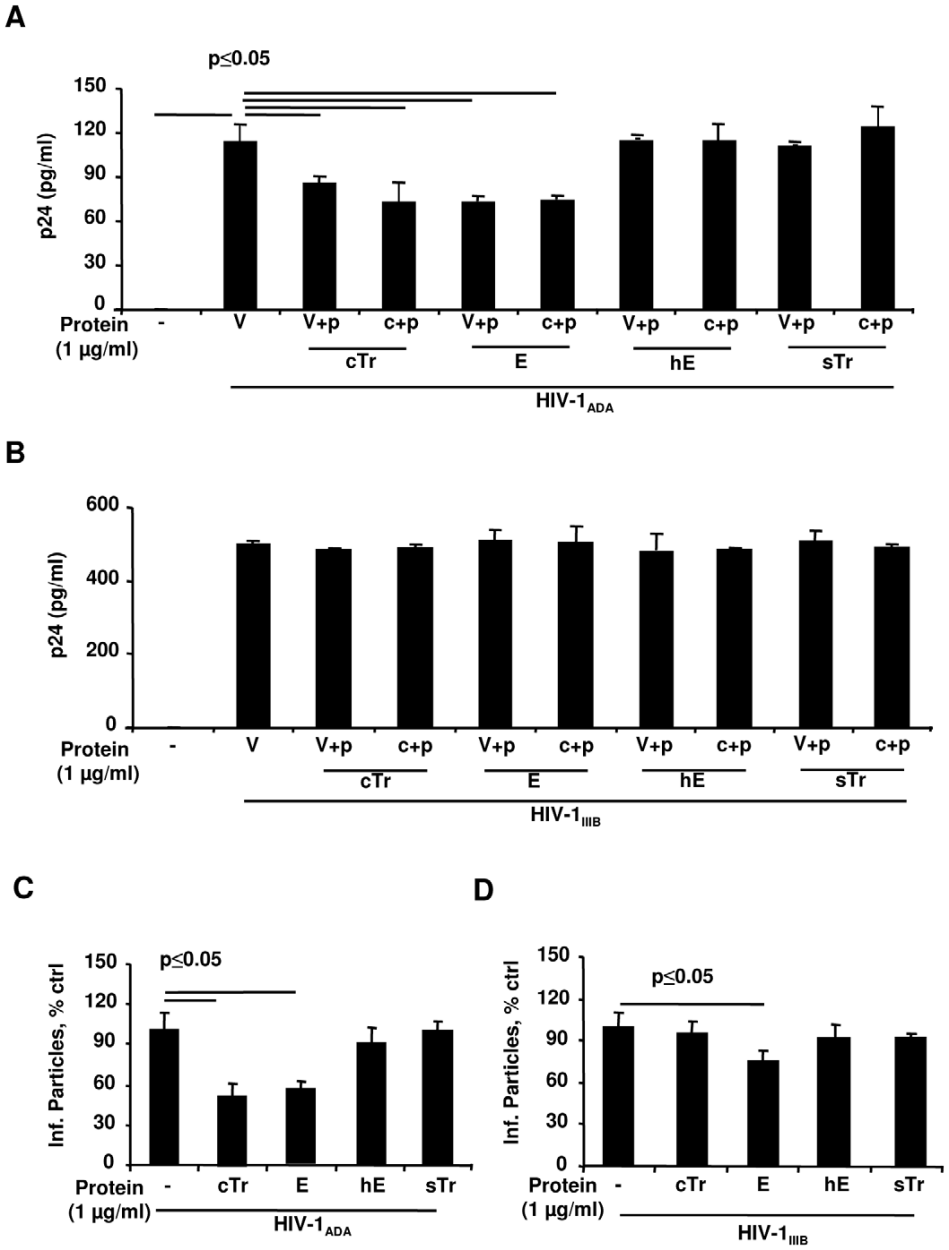
Unmodified N-terminus of elafin is critical for anti-HIV-1, but not antiprotease, activity. For attachment assay (*A,B*), 100 TCID_50_ of R5-HIV-1_ADA_ (*A*), X4-HIV-1_IIIB_ (*B*), and HEC-1A cells were incubated with media alone or cTr, E, hE, or sTr at 1 µg/ml for 1 h at 37°C. Following the incubation, cells were repeatedly washed with PBS. Subsequently, cells not pretreated with the proteins received either medium alone (−) or untreated HIV-1 (V), or HIV-1 pretreated with Tr or E (V+p), whereas protein-treated cells (c+p) received HIV-1 alone (V). Following 1.5 h of incubation at 37°C, viral inoculum was removed and cells were repeatedly washed (4×) and lysed (1% Triton X-100 for 45 min at 37°C). Cell lysates were harvested and the amount of total cell lysate-associated p24 was determined by ELISA. The data are representative of one of three independent experiments performed in triplicate and shown as the mean ± SD of p24 protein expressed as pg/ml. Statistical analysis was performed using ANOVA, with p values considered significant when p<0.05. For transcytosis (*C,D*), HEC-1A cells were grown as a tight polarized monolayer on a permeable polycarbonate support and pretreated apically with media or 1 µg/ml of Tr or E proteins for 1 h before the addition of medium or 10 ng p24 of R5-HIV-1_ADA_ (*C*) or X4-HIV-1_IIIB_ (*D*) for 8 h at 37°C to the apical chamber. Data are representative of one of three independent experiments performed in triplicate and are shown as the mean ± SD. Amount of infectious HIV-1 particles in the concentrated supernatants from basolateral chamber were quantitatively analyzed by testing β-galactosidase activity of TZM-bl cells, resulting in cells turning blue upon HIV-1 infection as described in Materials and Methods and elsewhere [46]. Data are expressed as percentage of infectious particles recovered in the presence of Tr or E compared to the percentage of virus recovered in the virus control alone, taken as 100%. Statistical analysis was performed using ANOVA or Student's *t* with p values considered significant when p<0.05. Concentrations of Tr and E used in these experiments were based on a dose-dependent anti-HIV-1 activity of the proteins that was reported earlier [30].

Next, to test anti-HIV-1 activity of E we utilized attachment/entry and trancystosis assays, since we previously demonstrated that cTr and especially its cleaved form, E, reduced HIV-1 attachment and transcytosis in HEC-1A cells [30]. Further, since structure-function parameters of E with anti-HIV-1 activity are unknown, and since we previously showed that hE, with modified N-terminus, did not inhibit VSV-GFP infection of HEC-1A cells, unlike cTr, with modified C-terminus [3], we utilized untagged and tagged Tr/E protein preparations to determine if N-terminus of E was important for anti-HIV-1 activity. In the attachment/entry assay, both HEC-1A cells and 100 TCID_50_ of R5-HIV-1_ADA_ and X4-HIV-1_IIIB_ were individually pretreated with medium or 1 µg/ml of Tr/E proteins for 1 h at 37°C to see if E could act separately through cells and HIV-1. Following incubation and repeated washing, cells were exposed to either medium alone or virus. Cells that were not initially pretreated with Tr/E proteins, received either medium, or untreated virus, as a positive control, or virus pretreated with proteins. Conversely, cells that were initially pretreated with the proteins, received only untreated virus. After additional incubation for 1.5 h at 37°C, p24 levels were determined in total cell lysates by ELISA. For transcytosis, HEC-1A cells were grown as a tight polarized monolayer and were pretreated apically with media or 1 µg/ml of Tr or E proteins for 1 h before the addition of medium or 10 ng p24 of R5-HIV-1_ADA_ or X4-HIV-1_IIIB_ for 8 h at 37°C. Transcytosed infectious HIV-1 particles were quantitatively analyzed in the basolateral compartment supernatants by testing β-galactosidase activity of TZM-bl cells, resulting in cells turning blue upon HIV-1 infection, as described in detail in Materials and Methods. The results of anti-HIV-1 activity testing of the utilized Tr/E proteins demonstrated that, in contrast to cTr and E, hE and sTr did not exhibit anti-HIV-1 activities, as assessed in attachment and transcytosis assays (Fig. 2A–D). Specifically, the attachment of R5-HIV-1_ADA_ strain was significantly reduced, when either cells or the virus were pretreated with cTr and E, but not with sTr or hE (Fig. 2A). This observation was also in line with results of transcytosis assay (Fig. 2C and D). Unlike the attachment/entry results, however, E also inhibited transcytosis of X4-HIV-1_IIIB_ (Fig. 2D). Notably, Eh had similar HIV-1 inhibitory activity in the attachment/entry assay, where pretreatment of R5-HIV-1_ADA_ with 1 µg/ml of the protein resulted in reduction in p24 from 84.59±16.54 to 49.55±21.55 pg/ml, p<0.041; or in the case of cells pretreatment, from 84.59±16.54 to 58.92±12.08 pg/ml, p<0.05, n = 4 in each case, Student's *t* test. Additionally, testing of metabolic activity of cells revealed no differences in viability and metabolic activity among the groups (data not shown). Importantly, these results also indicate that unmodified N-terminus of E is critical for anti-HIV-1 activities of the protein, but not for its antiprotease activity. Further, sTr which had significantly elevated antiprotease activity lacked anti-HIV-1 activity.

### Cellular localization of exogenous elafin in genital epithelial cells

**Figure 3:**
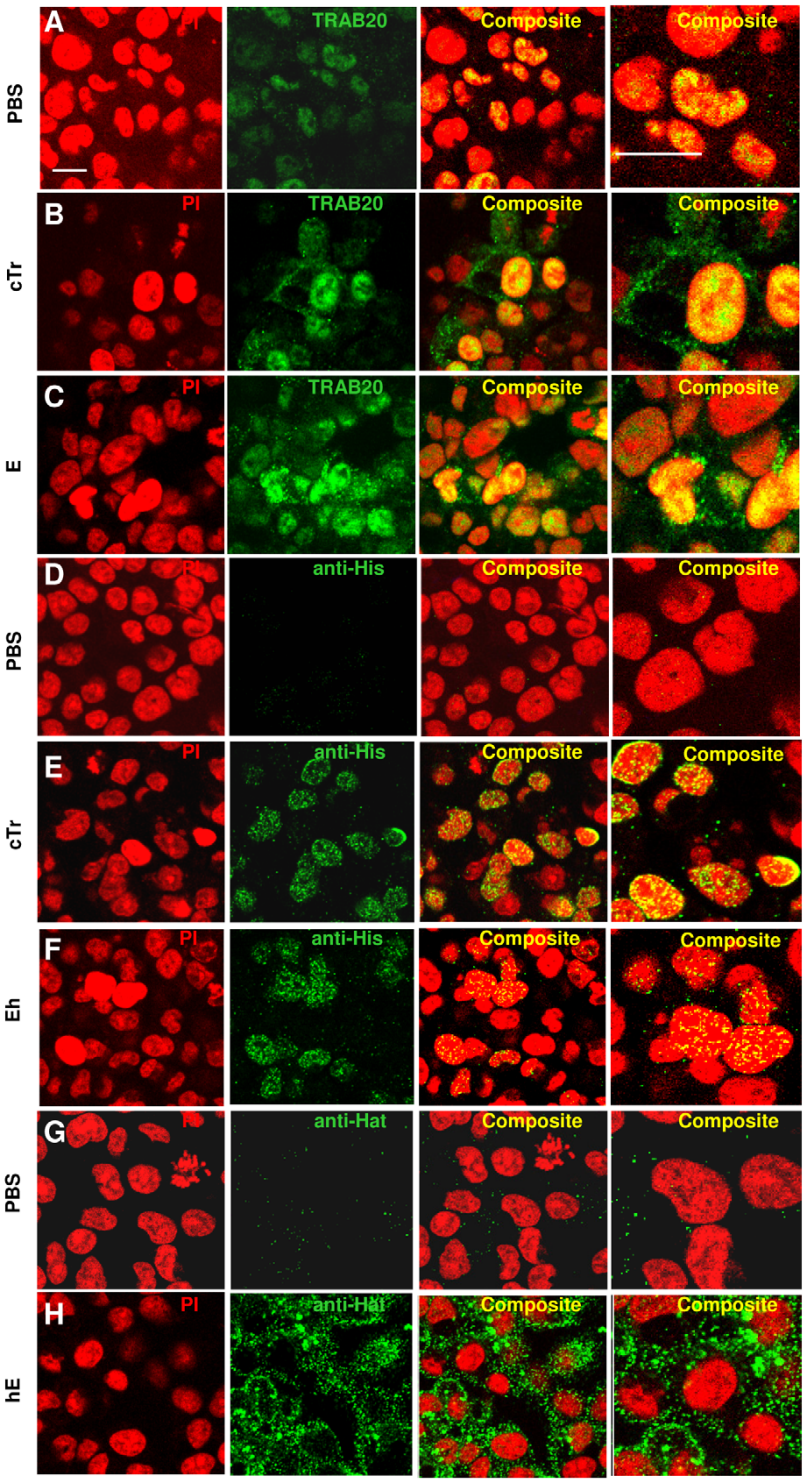
Cellular localization of exogenous elafin in genital epithelial cells. Immunofluorescence staining of cellular distribution of 1 µg/ml of human Tr or E (green) proteins, detected using TRAB2O (*A,B,C*), His-tag- (*D,E,F*), and HAT-tag-specific antibodies (*G,H*). Nuclei were visualized with propidium iodide (PI) as red. Representative staining of three different experiments is shown for Tr or E proteins at 1 h post exposure and merged image shows human Tr or E visualized as yellow in composite panels. Scale bar, 20 mm.

Since E treatment reduces HIV-1 attachment/entry and transcytosis in HEC-1A cells, and since we previously demonstrated that Tr/E modulated polyI:C-induced antiviral and pro-inflammatory responses [3], we hypothesized that E may also modulate HIV-1-triggered responses at the intracellular level. To ensure intracellular localization of tested Tr/E, we next determined the cellular distribution of exogenously added Tr/E at 1 µg/ml to HEC-1A cells for 1 h at 37°C using immunofluorescence confocal microscopy. Images from control HEC-1A cells treated with PBS and stained with anti-Tr/E (TRAB20) antibodies were merged with images from propidium iodide-stained nuclei. Merged images showed a low level of endogenously expressed E in the nuclei of HEC-1A cells (Fig. 3A). Interestingly, exogenous E, added as cTr or E, was rapidly taken up by cells (within 1 h) and accumulated to high levels in the nucleus (Fig. 3B & 3C). In order to clearly distinguish exogenous and endogenous E, we next used and tracked tagged-E with anti-His or anti-Hat antibodies. Control HEC-1 cells treated with PBS and stained with anti-His (Fig. 3D) or anti-Hat (Fig. 3G) antibodies were negative. HEC-1 cells treated with exogenous cTr and Eh and stained with anti-His were distributed largely to the perinuclear and nuclear region (Fig. 3E and F). Unexpectedly, hE that has an N-terminal Hat tag was localized near the periphery of the plasma membrane and not detected in the nucleus (Fig. 3H). Together these data indicate that low levels of endogenous E and exogenously added E bearing unrestricted N-terminus, rapidly enters the nucleus of HEC-1A cells. Further, the nuclear localization of E was associated with anti-HIV-1 responsiveness of HEC-1A cells. Moreover, since endogenous E in HEC-1A cells can be present intracellularly (Fig. 3A) and be secreted in response to bacterial (data not shown) and viral [3], [30] ligands, and since extracellular E can also enter HEC-1A cells, we propose that anti-HIV-1 activity of E (Fig. 2) can be a result of its autocrine/paracrine function in HEC-1A cells.

### Elafin inhibits IL-8 secretion in response to R5-HIV-1_ADA_ in HEC-1A cells

**Figure 4:**
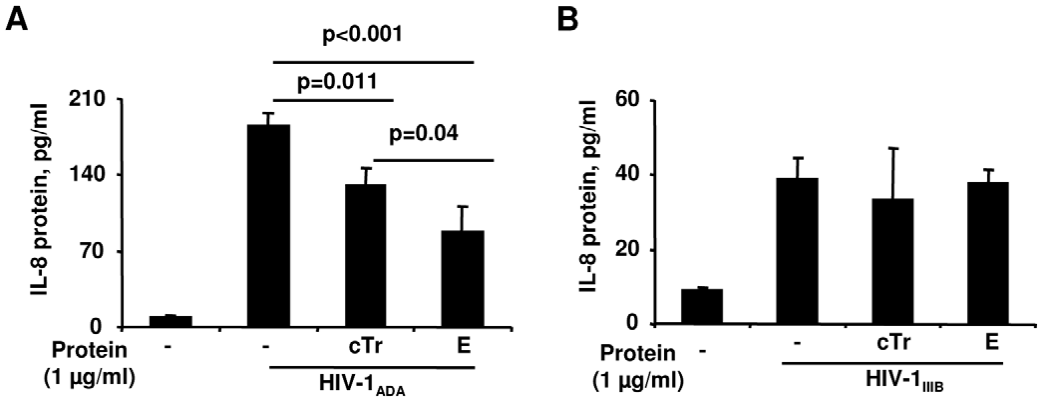
Elafin inhibits IL-8 secretion in response to R5-HIV-1_ADA_ in HEC-1A cells HEC-1A cells were grown as a polarized monolayer in a transwell system and incubated with media or 1 µg/ml of cTr or E for 1 h before the addition of 10 ng p24 of R5-HIV-1_ADA_ (*A*) or X4-HIV-1_IIIB_ strains (*B*) for 8 h at 37°C to the apical chamber. Levels of IL-8 protein were determined in basolateral compartment by ELISA and shown as the mean ± SD of pg/ml. Statistical analysis was performed using ANOVA, with p values indicated on a graph and considered significant when p<0.05.

HIV-1 interaction with genital ECs triggers the release of pro-inflammatory factors, including IL-8 and TNFα [48], [49]. Considering that previous studies demonstrated immunomodulatory properties of Tr/E against bacterial [34] and viral ligands [3], we hypothesized that E may affect HIV-1-induced secretion of IL-8 and TNFα from HEC-1A cells, implicated in STI/HIV-1 dissemination and pathogenesis [49]. HEC-1A cells were pretreated with medium alone or with 1 µg/ml of cTr, E, or hE proteins for 1 h at 37°C and then additionally stimulated with 10 ng of HIV-1 p24 for 8 h at 37°C, the conditions favoring HIV-1 attachment/entry and transcytosis. ELISA measurements demonstrated significantly lower levels of IL-8 in the basolateral compartment of HEC-1A cells after pretreatment with E or cTr, but not with hE (data not shown), following R5-HIV-1_ADA_ (Fig. 4A), but not X4-HIV-1_IIIB_ exposure (Fig. 4B). Levels of TNFα, however, were negligible and around the level of detection across the groups for both R5- and X4-HIV-1 virus-treated samples (data not shown). These results suggest that E treatment alters HIV-1-induced inflammatory responsiveness of HEC-1A cells.

### Elafin attenuates HIV-1-induced nuclear translocation of NF-κB and expression of several PRR genes in response to R5-HIV-1_ADA_

**Figure 5:**
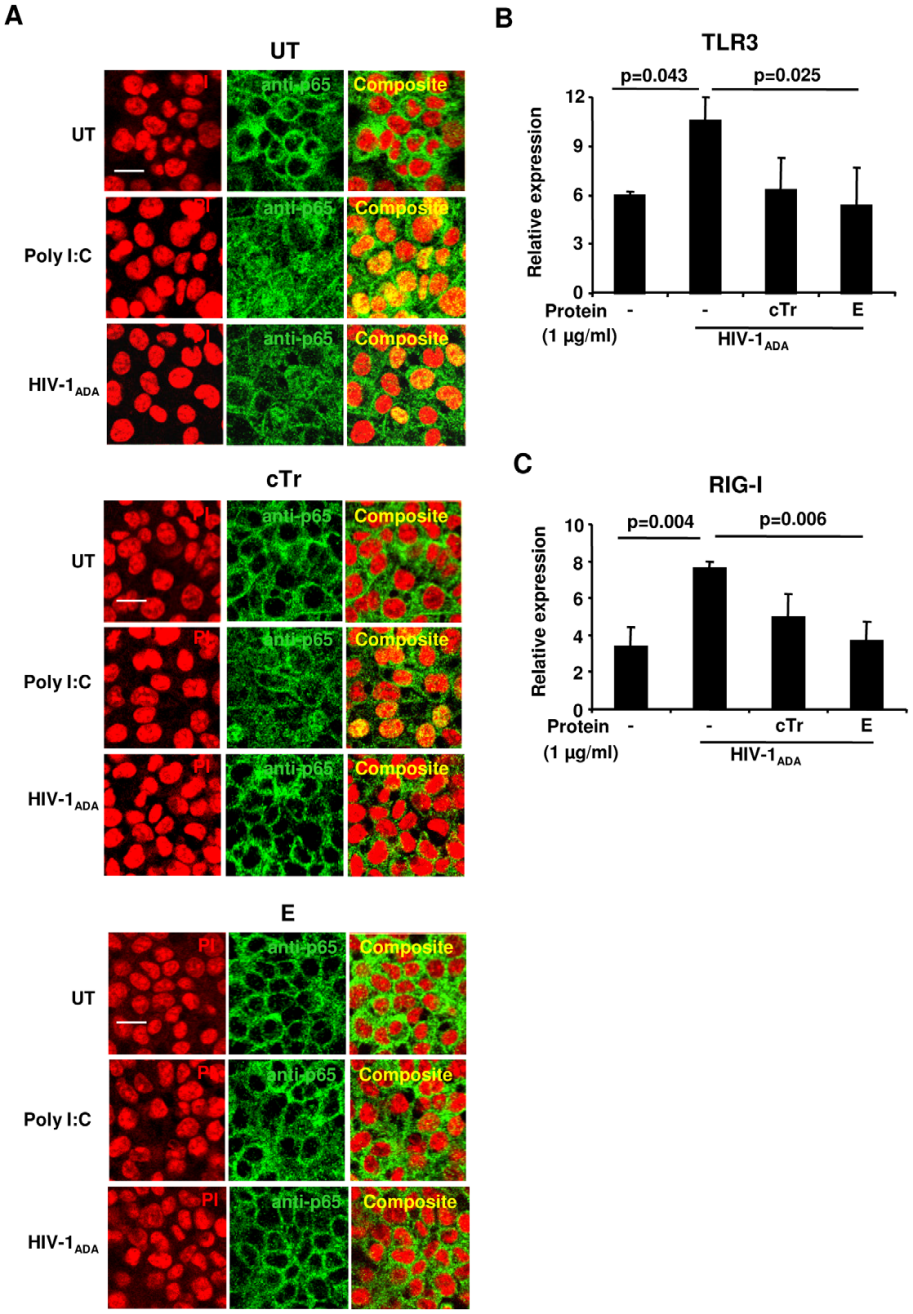
Elafin attenuates HIV-1-induced nuclear translocation of NF-κB and mRNA expression of TLR3 and RIG-I genes in response to R5-HIV-1_ADA_. Immunofluorescence staining of NF-κB/p65 and its nuclear translocation following either medium alone or polyI:C 25 µg/ml, or 10 ng of R5-HIV-1_ADA_ p24 treatment for 4 h in presence or absence of pre-treatment with 1 µg/ml of cTr or E proteins is demonstrated (*A*). Representative staining is shown for NF-κB/p65 (green) visualized by Alexa488, nuclear stain (red) visualized by propidium iodide (PI), and composite (yellow). Representative data set from three independent experiments is shown. Scale bar, 20 mm. TLR3 (*B*) and RIG-I (*C*) mRNA expression was assessed by harvesting total RNA and performing RT-qPCR at 8 h post treatment with 10 ng of R5-HIV-1_ADA_ p24. Values are normalized against 18S and presented as relative expression. Representative data set from three independent experiments is shown as the mean ± SD. Statistical analysis was performed using one-way analysis of variance (ANOVA), with p considered significant when p<0.05.

We recently demonstrated that polyI:C treatment of HEC-1A cells was associated with induced NF-κB activation, increased release of IL-8, and induced expression of innate viral sensors RIG-I and MDA5 [3]. Furthermore, all of these observations were attenuated in Tr/E-treated cells and associated with greater antiviral protection against VSV-GFP challenge, suggesting a link between Tr/E, antiviral resistance, immunomodulation, and viral sensing. Since extracellularly added E can enter into HEC-1A cells that is associated with reduced HIV-1 attachment and transcytosis, as well as modulated HIV-1-induced release of IL-8 in HEC-1A cells, we hypothesized that E might be influencing HIV-1-induced intracellular signaling pathways, including NF-κB as one of the regulators of pro-inflammatory factors expression [8]. To evaluate this, untreated (UT) HEC-1A cells were pretreated with medium/buffer alone or with 1 µg/ml of cTr, E, or hE proteins for 1 h at 37°C and then stimulated with 25 µg/ml per well of polyI:C or 10 ng of HIV-1 p24 for 4 h at 37°C, the conditions favoring HIV-1 attachment/entry, intake and transcytosis according to our preliminary experiments (data not shown), and NF-κB/p65 nuclear translocation was assessed subsequently. Confocal immunofluorescence microscopy revealed that compared with UT-cells, E or cTr treatment significantly inhibited polyI:C- or R5-HIV-1_ADA_-induced nuclear translocation of NF-κB (Fig. 5A). Notably, polyI:C challenge produced slightly greater visible effect than R5-HIV-1_ADA_ challenge and was used as a positive control for the experiment. Further, X4-HIV-1_IIIB_ did not cause any significant NF-κB/p65 nuclear translocation (data not shown). Interestingly, unlike E or cTr, exogenous hE treatment failed to block NF-κB nuclear translocation and produced imagery similar to UT cells (data not shown).

PRRs are upstream regulators of NF-κB activation and the expression of pro-inflammatory mediators [8], [50]. Considering our previous data linking modulatory effects of Tr/E on polyI:C-induced PRRs expression, activation of inflammatory responses, and increased antiviral protection [3], and that pro-inflammatory conditions increase HIV-1 transcytosis across ECs monolayer [51], we hypothesized that E can reduce HIV-1 attachment/entry and transcytosis through the modulation of PRRs expression as regulators of inflammatory events. Thus, we next examined the expression level of innate viral sensors implicated in HIV-1 recognition/pathogenesis [52] in the presence of either E, cTr, or hE and following HIV-1 challenge. Results showed that cells pretreated as in transcytosis assay with E and cTr, but not hE (data not shown), had lower mRNA expression of TLR3 and RIG-I following R5-HIV-1_ADA_, compared to virus alone group (Fig. 5B and C) 8 h post treatment with 10 ng of HIV-1 p24. In contrast to R5, no differences in TLR3 or RIG-I expression were observed for X4-HIV-1_IIIB_ (data not shown). Overall, the data indicate that HEC-1A cells, pretreated with E, showed significantly lower level of induction of innate sensors in response to viral stimulation.

### Genital ECs from HIV-resistant (HIV-R) commercial sex workers (CSWs) have significantly lower expression of several PRRs

**Figure 6:**
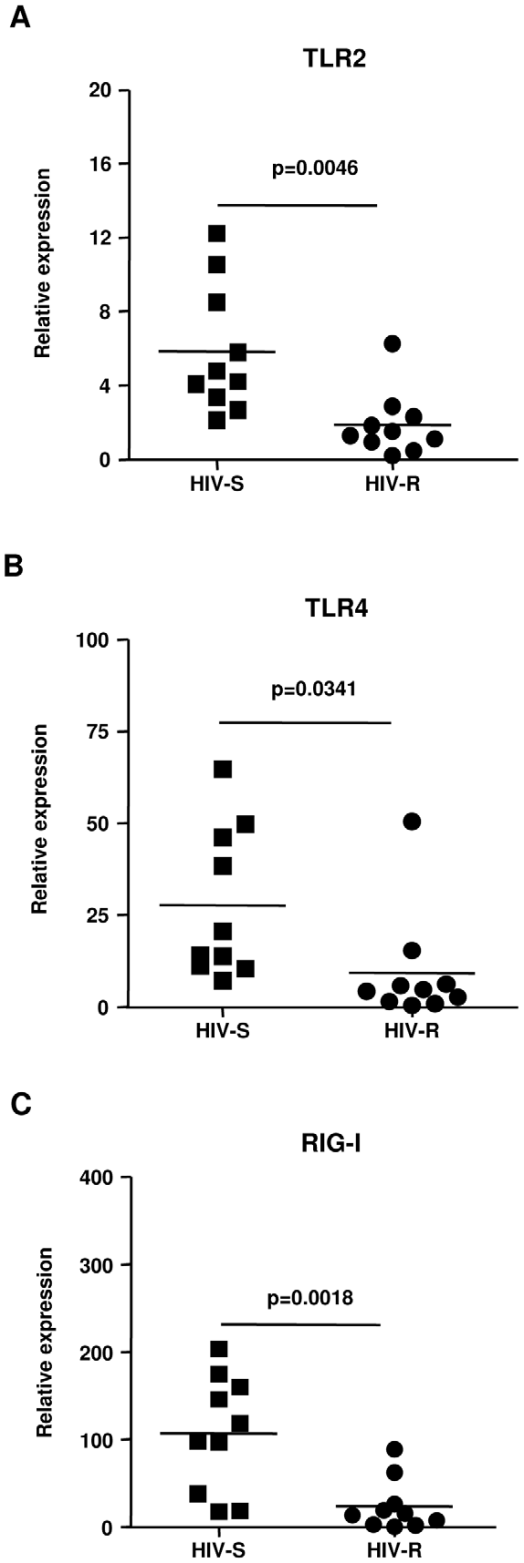
Genital ECs from HIV-resistant (HIV-R) commercial sex workers (CSWs) have significantly lower mRNA expression of TLR2, TLR4, and RIG-I. Relative mRNA expression levels of TLR2, TLR4, and RIG-I in cervical ECs of HIV-S (▪, N = 10) and HIV-R (•, N = 10) were assessed by RT-qPCR. The relative quantity of expression of these genes was normalized against 18S. The data are shown as the mean ± SD. Statistical analysis was performed using ANOVA, with p considered significant when p<0.05.

Given our *in vitro* data (Fig. 5), we investigated whether HIV-R CSWs, who remain HIV-free and have higher Tr and E in their CVLs [22], [30], also had reduced expression of innate viral sensors in genital ECs. Quantitative RT-PCR of cervical ECs revealed that HIV-R CSWs indeed had significantly reduced mRNA levels of TLR2, 3, 4 and RIG-I compared to susceptible (HIV-S) CSWs (Fig. 6). Collectively, our data indicate that HIV-1 resistance may be associated with increased levels of E-mediated decreased expression of mucosal innate PRRs on genital ECs at the portal of viral entry.

## Discussion

Here, we comparatively evaluated different Tr and E protein preparations with respect to their HIV-1 inhibitory, immunomodulatory, and antiprotease activities. To determine how E is mediating anti-HIV-1 effects in genital HEC-1A cells, we assessed its intracellular localization and effect on viral recognition and mounting HIV-1-driven inflammatory responses using HIV-1 non-canonical receptor-bearing genital epithelial HEC-1A cells, as well as tagged and untagged Tr and E proteins. Our results revealed that both E and Tr could function as autocrine/paracrine factors in HEC-1A cells, but that it was E, and not Tr, that likely acted against HIV-1. Further, we observed that anti-HIV-1 activity of E was highly associated with autocrine/paracrine, but not antiproteolytic, property of E and also depended on its unmodified N-terminus. Specifically, we showed that E with unmodified N-terminus attenuated R5-HIV-1_ADA_-induced NF-κB/p65 nuclear translocation, as well as levels of IL-8 secretion and expression of PRRs TLR3 and RIG-I that were associated with reduced HIV-1 attachment and transcytosis in HEC-1A cells. Importantly, our results also showed that elevated levels of Tr/E in CVL of HIV-R CSWs were associated with significantly lower mRNA levels of TLR2, 3, 4, and RIG-I in the genital ECs of this cohort, suggesting a link between Tr/E, HIV resistance and modulated viral recognition in the FGT. Collectively, we propose that E-mediated altered intracellular innate activation is most likely contributing to the HIV-R property of these subjects.

The antiprotease activity of Tr/E depends on the four-disulfide core structure, or the WAP motif, that is localized to the C-terminal inhibitory domain of Tr/E_ [53], [54] and is also present in SLPI_ [55], [56]. Our finding that all Tr and E preparations used in this study were nearly equally active against HNE allowed for comparative assessment of anti-HIV-1 and immunomodulatory properties of Tr/E, despite the structural and manufacturing differences present among the tested proteins. Since not all the tested Tr and E protein preparations exhibited anti-HIV-1 activity, this provided the first experimental evidence that antiprotease and anti-HIV-1 activities of E do not correlate with each other. Our data were supported by earlier reports demonstrating that A62D/M63L, a Tr variant lacking antiprotease properties, had normal antibacterial and antifungal activities _[31], and also by the fact that a mutant of SLPI lacking antiprotease activity demonstrated intact anti-HIV-1 activity [57]. Collectively, these observations highlight that E exerts antiviral effects through mechanisms and structures distinct from those mediating its antiprotease function.

Our results demonstrated that in HEC-1A cells endogenous Tr/E were present either intracellularly, including the nuclear area, or secreted extracellularly in response to bacterial and viral ligands (data not shown) [30]. We further showed that extracellular Tr/E could enter HEC-1A cells within 1 h of exposure and localize within nuclear/perinuclear areas, similar to endogenous proteins. Since anti-HIV-1 inhibitory activity of Tr/E was highly associated with the proteins' entry into the cells, and not their antiprotease activity, we conclude that Tr/E in their defense against HIV-1 were likely acting through autocrine/paracrine function; we believe the latter function is separate and possibly complimentary to other virus- and cell surface receptor-mediated anti-HIV-1 activities of the tested Tr/E proteins.

Our data showed that E and cTr/Eh, but not sTr or hE, exhibited anti-HIV-1 activity. We propose that most likely it is the presence of E, but not Tr, that is important for antiviral activity of the tested Tr and E proteins. Based on the collective findings that cTr is a mixture of both Tr and E [3], that sTr did not show any HIV-1 inhibition, and that anti-HIV-1 activity of E was 130 times more potent than Tr [30], we suggest that it is the presence of E in cTr that highly likely is attributing to anti-HIV-1 activity of cTr. Moreover, our recent observations on the limited antiviral activity of sTr against vesicular stomatitis virus (VSV-GFP) [3] would also support this notion.

Furthermore, that E and Eh, but not hE, exerted HIV-1 inhibitory activity strongly suggested that tagging of the N-terminus most likely blocked the antiviral property of hE, as well as its ability to act in autocrine/paracrine manner and to enter nuclear/perinuclear area following cell pretreatment. These findings indicated that the N-terminus of E was critical for anti-HIV-1 activity, and proposed an alternative to the general conception that cationic property of Tr/E might be critical for antiviral activities. Indeed, our bioinformatics analysis of isoelectric points (GENETYX, version 8.2.0) revealed that E (isoelectric point 8.07) is less cationic than its precursor Tr (isoelectric point 8.97).Yet, E is 130 times more potent than Tr with respect to anti-HIV-1 activity, clearly indicating that cationic property is not predetermining anti-HIV-1 activity of E [30]. The antimicrobial property of several other cationic antimicrobials has also been attributed to the N-terminal domain, as was shown for lactoferrin [58], [59]. __Additionally, our earlier studies showing the lack of antiviral effect of hE against VSV-GFP challenge also support our current findings [3].

NF-κB is one of the transcription factors regulating production of pro-inflammatory cytokines, and nuclear translocation of NF-κB is directly associated with secretion of TNFα and IL-8 [60] _as well as HIV-1 pathogenesis and replication [61], [62], [63]. It was reported that SLPI can affect NF-κB nuclear translocation and influence gene regulation [64]; on conjecture, we proposed that, being a functional homolog of SLPI, E most likely performs its anti-HIV-1 effect through modulation of NF-κB nuclear translocation and regulation of pro-inflammatory and innate sensing genes. However, the mechanism of direct virus- or cell surface receptor-mediated, anti-HIV-1 activity of E might be different. Although Tr/E secretion can be elevated in the presence of inflammatory stimuli, our earlier study [3] indicated that, in response to polyI:C treatment, exogenous E treatment can also enhance inflammatory stimuli secretion, albeit at higher protein concentrations, which indicates a close feedback loop between these two circuitries.

Our findings that apical treatment of HEC-1A cells with E under challenge of R5-HIV-1_ADA_ resulted in reduced secretion of IL-8 into the basolateral compartment might have important implications for activation of additional HIV-1 target cells located in the genital submucosa. This could limit local inflammation and the attraction of HIV susceptible cells. This notion is also supported by the fact that elevated levels of Tr/E in CVL of HIV-R CSWs are associated with higher anti-HIV-1 activity *in vitro* [30] and mucosal resistance to HIV-1 infection *in vivo*.

HIV-1 acquisition and disease progression are associated with immune activation _[52], [65] and dysregulation of expression and responsiveness of viral sensors [66], [67], [68], [69], [70]. Specifically, we recently showed that chronic untreated HIV-1 infection was associated with aberrant expression and responsiveness of TLR2, 3, 4, 6, 7/8 in PBMCs [66], [67]_. Further, Sooty Mangabeys, a natural host of simian immunodeficiency virus (SIV), show no signs of immune activation/inflammation, despite high systemic viraemia _[15]. In contrast, a non-natural and SIV-susceptible reservoir, African Green Monkeys, show marked inflammatory responses and disease progression. These observations suggest that HIV-induced alterations in innate immune responsiveness may initiate and perpetuate immune activation, dysfunction, and inflammation that manifest in HIV-1/AIDS pathogenesis and disease acceleration. Since resistance to HIV-1 has been associated with “immune quiescence” and decreased cellular activation [71] on the one hand, and robust, but transient IRF1 antiviral responses [72], in peripheral blood-derived monocytes on the other hand, it appears that modulation of pathogen recognition and controlling untimely or unnecessary inflammatory responses can be beneficial and pivotal in determining the fate of HIV-host encounter and disease establishment. Considering that Tr/E were shown to down-modulate antibacterial [34], [35] and antiviral innate immune responses [23], without negatively affecting antimicrobial protection [3], one can argue that it might be beneficial in the case of HIV-1 to have Tr/E at mucosal surfaces to modulate innate immune responsiveness while increasing antiviral protection.

Importantly, we found that E not only influenced HIV-1 infection, but also affected the relevant intracellular molecular circuitry as demonstrated by reduced IL-8 secretion, nuclear translocation of NF-κB, and decreased mRNA expression of several innate viral sensing receptors, for which specific mechanisms remain to be determined. These data are reminiscent of our earlier findings in response to viral dsRNA mimic, polyI:C, showing that Tr/E mediated increased polyI:C-driven antiviral protection that was associated with reduced pro-inflammatory factors as well as lower expression levels of RIG-I and MDA5, and NF-κB activation [3]. Our data from cervical ECs from CSWs further support the argument that moderated, or lower, expression of local innate PRRs in the context of repeated viral exposures might be a predetermining factor in resistance to HIV-1 mucosal transmission, and that E may play a direct role in these events. Although the purity of cervical ECs was not verified here, thus potentially allowing for the presence of other cell types in the cervical samples, these data demonstrate significantly reduced immune activation of cervical cells from HIV-R CSWs compared to the susceptible controls. Further studies are clearly necessary to more specifically examine PRR expression and regulation in all cells and tissues of the FGT tract in HIV-R cohort and their controls.

It remains to be determined how exactly intranuclear localization of Tr/E could interfere with HIV-1 adsorption/entry and transcytosis through ECs. However, it is plausible that Tr/E could be acting at both levels simultaneously, but only Tr/E proteins with unmodified N-terminus are capable of both interacting with HIV-1 binding/entry receptors as well as acting inside the nucleus. For the nuclear localization, we propose that this mechanism likely involves Tr/E modulation of inflammatory responses induced upon HIV-epithelial cell interaction. Such a mechanism is supported by an earlier study showing that transcytosis of cell-free HIV-1 across HEC-1A was enhanced in pro-inflammatory conditions via as an yet unidentified pathway [51]. Arguably, such inflammatory events may trigger or bolster HIV-1 attachment and trancytosis by inducing the expression or rearrangement of HIV-binding/entry receptors or innate viral sensors that would facilitate viral adsorption, uptake and passage through epithelial monolayer.

Even though several HIV-1 binding/entry receptors, including syndecans, galactosylceramide, mannose moieties [6], have been implicated in HIV-1 transcytosis, the precise step-by-step mechanism of HIV-1 transcytosis is still poorly understood, as well as factors controlling it. Given the multitude of the receptors involved, it can be anticipated that a close interplay amid these receptors would predetermine the magnitude and efficiency of HIV-1 passage through ECs. This speculation can be supported by a recent observation in CD4+ T cells showing that an interaction between non-canonical α4β7 integrins with HIV-1 gp120 facilitated further interaction between gp120 and other cellular HIV-1 receptors, CCR5 and CD4, ultimately resulting in CD4+ T cell activation and increased cell-cell HIV-1 transfer [73]. Thus, the proposed antiviral mechanism of Tr/E may involve the counterbalancing effect of Tr/E on cell activating events that could occur either through binding to DNA, or reducing phosphorylation and degradation of NF-kB inhibitors, or by limiting the availability and expression of viral PRRs and attachment/entry HIV receptors that may ultimately lead to reduced cell activation, less pro-inflammatory milieu, and reduced cellular susceptibility to HIV-1 attachment/entry. Our recent observations that reduced polyI:C-triggered secretion of IL-8 and TNFa, as well as NF-kB activation and the expression of innate viral sensors in the presence of Tr/E were associated with increased antiviral protection following VSV-GFP challenge [3] further support the argument that reduced cellular immune activation may play a beneficial role in antiviral defense.

In conclusion, although we realize that our *in vitro* experiments only partially recreated the conditions present in the genital tract of CSWs, our study highlighted potential relationships/mechanisms and targets of E's antiviral activities in genital ECs that could be critical in reducing susceptibility to HIV-1 mucosal infection and potentially used in microbicide trials. While presented evidence may point toward a promising anti-HIV-1 activity of E alone, its ultimate protective anti-HIV-1 effect might be more evident *in vivo* at mucosal sites, acting in synergy with many other innate effector proteins, including defensins, cathelicidins, lactoferrin, and SLPI.
